# 

**Published:** 1891-08

**Authors:** 


					﻿Vol. XI.
AUGUST, 1891.
No. 8.
THE
pOMIOPATHlC pHY^lClAJfl,
A Monthly Journal of Homoeopathic Materia Medica
and Clinical Medicine.
“He is a freeman whom truth makes free, and all are slaves beside."
“Seek the Truth: come whence it may, cost what it will."
EDITED AND PUBLISHED BY
WALTER M. JAMES, M. D.,
AND
George h. Clark, m. id.
PHILADELPHIA :
No. 1185 ST’RTTCK STREET.
LONDON
ALFRED HEATH & CO., Sole Agents for Great Britain,
.114 Ebury Street, Eaton Square.
SS- Yearly Subscription, $2.60, invariably in advance. Single numbers, 26 cts.
Entered at the Philadelphia Paet-Office aa Seoond- ClaM Mail Matter.
				

